# Potential Short-Term Air Pollution Effects on Rheumatoid Arthritis Activity in Metropolitan Areas in the North of Italy: A Cross-Sectional Study

**DOI:** 10.3390/ijerph18168490

**Published:** 2021-08-11

**Authors:** Francesca Ingegnoli, Tania Ubiali, Tommaso Schioppo, Valentina Longo, Antonella Murgo, Orazio De Lucia, Ennio Giulio Favalli, Simona Iodice, Valentina Bollati, Roberto Caporali

**Affiliations:** 1 Division of Clinical Rheumatology, ASST Pini-CTO, 20122 Milano, Italy; tania.ubiali@gmail.com (T.U.); Tommaso.schioppo@unimi.it (T.S.); vale.longo94@gmail.com (V.L.); Antonella.Murgo@asst-pini-cto.it (A.M.); oraziodelucia@alice.it (O.D.L.); ennio.favalli@gmail.com (E.G.F.); roberto.caporali@unimi.it (R.C.); 2 Department of Clinical Sciences & Community Health, Research Center for Adult and Pediatric Rheumatic Diseases, Research Center for Environmental Health, Università degli Studi di Milano, 20122 Milano, Italy; valentina.bollati@unimi.it; 3 EPIGET–Epidemiology, Epigenetics and Toxicology Lab, Department of Clinical Sciences & Community Health, Università degli Studi di Milano, 20122 Milano, Italy; simona.iodice@unimi.it

**Keywords:** rheumatoid arthritis, air pollution, particulate matter, disease activity

## Abstract

Rheumatoid arthritis (RA) flare is related to increased joint damage, disability, and healthcare use. The impact of short-term air pollution exposure on RA disease activity is still a matter of debate. In this cross-sectional study, we investigated whether short-term exposure to particulate matter (PM)_10_, PM_2.5_, nitrogen dioxide (NO_2_), and ozone (O_3_) affected RA disease activity (DAS28 and SDAI) in 422 consecutive RA residents in Lombardy, North of Italy. Air pollutant concentrations, estimated by Regional Environmental Protection Agency (Lombardy—Italy) at the municipality level, were used to assign short-term exposure from the day of enrolment, back to seven days. Some significant negative associations emerged between RA disease activity, PM_10_, and NO_2_, whereas some positive associations were observed for O_3_. Patients were also stratified according to their ongoing Disease-Modifying anti-Rheumatic Drugs (DMARDs) treatment: no DMARDs (*n* = 25), conventional synthetic DMARDs (*n* = 108), and biological or targeted synthetic DMARDs (*n* = 289). Therapy interaction seemed partially able to influence the relationship between short-term air pollution exposure and RA disease activity (PM_2.5_ levels and DAS28 at the day of the visit-O_3_ levels and disease activity scores for the seven days before the evaluation). According to our results, the impact of short-term air pollution exposure (seven days) minimally impacts disease activity. Moreover, our study suggests therapy could alter the response to environmental factors. Further evidence is needed to elucidate determinants of RA flare and its management.

## 1. Introduction

Rheumatoid arthritis (RA) is an autoimmune disease and it is mainly characterized by inflammatory joint involvement, potentially leading to progressive disability. RA is considered a global public health challenge with almost 20 million prevalent cases, 1.2 million incident cases, and 3.4 million disability-adjusted life years [1].

Despite considerable advances in RA pathogenesis, both genetic and environmental factors have not been fully clarified. Environmental exposures, such as cigarette smoking, silica dust, and mineral oil, can promote oxidative stress, increase inflammation, and induce bronchus-associated lymphoid tissue to produce anti-citrullinated protein antibodies (ACPA) [2,3,4,5,6]. The role of long-term exposure to air pollution in RA development was investigated in several studies with controversial results [7,8,9,10,11,12]. In a meta-analysis published in 2020, long-term exposure to ozone (O_3_) and living near traffic roads were reported to increase the risk of RA, while other pollutants, such as particulate matter (PM), did not seem to have an impact [13].

Once RA is diagnosed, according to current recommendations, patients should reach and maintain disease remission by applying current recommendations for tight control and treat-to-target strategies [14]. Disease flares should be avoided as much as possible to limit disease progression and activity, which can increase disability, healthcare use, costs, and therefore impair health-related quality of life [15]. To date, there are no available data to predict disease flare in RA patients. So far, only a few studies have been published about the effects of short-term pollution exposure on RA disease activity. One is based on the Kuwait Registry for Rheumatic Diseases, which described the detrimental effects of short-term sulfur dioxide (SO_2_) and nitrogen dioxide (NO_2_) exposure on RA disease activity, while no correlation was found for PM_10_, O_3,_ and carbon monoxide (CO) [16]. Another one was conducted in China (Hefei region): the exposure to a high concentration of PM_2.5_ and NO_2_ was related to hospital readmission within one year after the last discharge in RA patients [17]. Recently, a longitudinal study in the Veneto region (Italy) reported a significant association between disease reactivation and medium-term air pollution exposure (30-days and 60-days before the assessment) [18].

In this study, the effects of short-term exposure (seven days) to air pollutants (PM_10_, PM_2.5_, NO_2,_ and O_3_) on RA disease activity in patients referring to a rheumatology unit in Milan (north of Italy) were investigated.

## 2. Materials and Methods

This cross-sectional, single-center, no-profit study was conducted between January and June 2018 at the Division of Clinical Rheumatology of G. Pini Hospital in Milan, University of Milan, Italy. The local ethics committee “Comitato Etico Milano Area 2” approved this study (approval code 17_2018). Informed written consent was obtained from all subjects.

All consecutive patients (aged > 18 years) referred to our center with a diagnosis of RA were enrolled in the study. RA was defined according to the American College of Rheumatology (ACR) and/or 2010 ACR/European League Against Rheumatism classification criteria [19,20]. Only patients resident in Lombardy (a region located in the north of Italy) with a disease duration longer than three months were considered eligible for the study. Patients with overlap syndromes (e.g., RA and systemic lupus erythematosus-SLE) were excluded.

For each patient, data on disease characteristics and disease activity were collected at enrolment. Moreover, data on pollutants exposure were obtained from the archives of the Regional Environmental Protection Agency (ARPA Lombardia).

At enrolment, the following information was collected: demographic and clinical data, disease activity (DAS28-CRP: disease activity score on 28 joints with C-reactive protein; SDAI: simplified disease activity index), and ongoing treatments. Moreover, physician’s and patient’s disease activity global assessments (PhGA and PaGA), tender and swollen joint counts (TJC and SJC), and patient’s global health (GH) were collected and analyzed separately.

For pollution exposure, daily mean PM_10_, PM_2.5_, NO_2,_ and O_3_ concentrations were retrieved from the Open Data Lombardy Region (https://www.dati.lombardia.it, accessed 10 August 2021) database, which contains daily estimates of municipal aggregate values calculated by the Regional Environmental Protection Agency (ARPA Lombardy). During the period analyzed in this study, no incidents that could influence emissions were reported. The assessment of pollutant concentrations is based on the ARIA Regional Modelling (www.aria-net.it, accessed 10 August 2021), a chemical–physical model of air quality that simulates the dispersion and chemical reactions of atmospheric pollutants. It integrates the data measured from the monitoring stations of the ARPA Lombardy air quality network, meteorological data, emissions, concentrations at the beginning of the simulation period, and trends in adjacent areas, covering the whole Lombard territory with a grid of 1 × 1 km^2^ cells, providing daily mean estimates available from the website at municipality resolution [21].

Each patient was assigned the daily concentration of each pollutant at the municipality in which they live, the day of evaluation, and each of the 7 days previous (i.e., from day 0 to day-7), we also calculate the mean of one week before the enrolment (i.e., week-1 is the mean of the day 0 to day-7). The variability of exposures is therefore due to spatial and temporal variations in exposure to pollutants among the study participants.

### Statistical Analysis

Descriptive statistics were performed on all variables. Continuous variables were expressed as the mean ± standard deviation (SD) or as the median with first-, and third-quartile (Q1–Q3), as appropriate. Categorical data were reported as frequencies with percentages. Multivariable linear regression models were performed to identify the day of PM_10_, PM_2.5_, NO_2,_ and O_3_ independently associated with DAS28, SDAI, GH, PhGA, PaGA, TJC, and SJC.

Models were adjusted for all parameters that could influence disease activity/flare: radiological damage, smoking habits, seropositivity for rheumatoid factor and/or ACPA, ongoing therapy with Disease-Modifying anti-Rheumatic Drugs (DMARDs) (no DMARDs, conventional synthetic-csDMARDs, targeted synthetic-tsDMARDs, biological-bDMARDs), use of steroids, age at examination, and disease duration. Each model was tested for normality and linearity. Departure from linearity was examined graphically and assessed by testing the null hypothesis that the coefficient of the second spline was equal to zero. Best model selection was based on the minimization of the Akaike information criterion and maximization of the explained variance of the model. All disease activity outcomes were log (base e) transformed to achieve normal distribution of residuals.

The potential effect of the therapy was investigated, adding an interaction term between pollutants and therapy in each model. When the interaction term resulted significantly (*p* < 0.05), the association between pollutant and outcome was investigated in each subgroup of therapy. *β* coefficients (the degree of change in the outcome variable for every 1-unit of change in the predictor variable) were reported for 10 µg/m^3^ increments of PM_10_, PM_2.5_, O_3,_ and NO_2_ concentrations.

In a sensitivity analysis, the window of exposure was enlarged to 14 days before the enrolment. The mean of 2 weeks before the enrolment (from day 0 to day-14) was also calculated. All changes in observed outcomes were associated with exposure within the first week before enrolment, so only results referring to 7 days before the visit for all the pollutants were considered.

All statistical analyses were performed using SAS 9.4 (SAS Institute, Cary, NC, USA).

## 3. Results

### 3.1. Patient Characteristics and Exposure Assessment

A total of 422 consecutive RA patients were enrolled with a mean age of 58.2 years and a mean disease duration of 16.1 years. The other demographic data and disease characteristics are reported in Table 1.

RA patients were stratified into three subgroups according to ongoing treatment: 25 subjects were without ongoing DMARDs (e.g., drug washout for side effects or comorbidities, long-standing remission, programmed therapy switch); 108 subjects were treated with csDMARDs, and 289 patients were treated with b/tsDMARDs. Based on the main demographic and clinical characteristics, the three groups were similar, except for smoking habits and positivity for RF and/or ACPA, which were higher in the b/tsDMARDs group. Disease activity indexes were similar in the 3 groups, except for PhGA (*p* = 0.05) and TJC (*p* = 0.036) that were higher in patients without ongoing therapy (no DMARDs).

**Figure 1:**
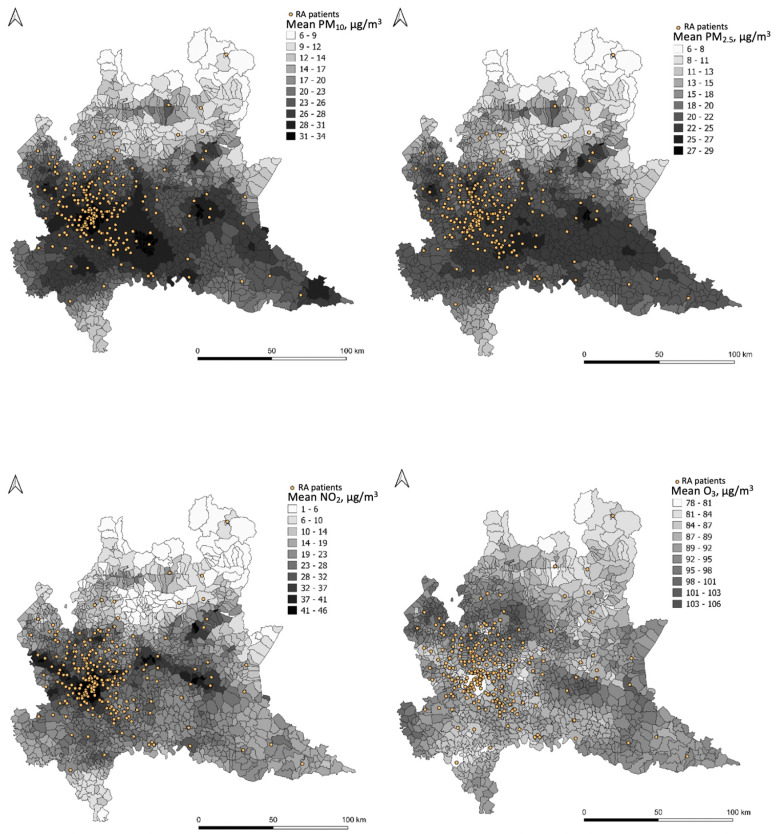
Graphical representation of PM_10_, PM_2.5_, NO_2_, and O_3_ concentration levels.

The daily concentrations of PM_10_, PM_2.5_, O_3,_ and NO_2_ at patients’ municipalities are reported in the Appendix A (Table A1, Table A2, Table A3 and Table A4). A map representation for each pollutant (PM_10_, PM_2.5_, NO_2_, O_3_) is reported in Figure 1. Each map reports the mean value, over the entire study period, for each municipality and the exact location of every participant of the study georeferenced to their residential address.

#### RA–Rheumatoid Arthritis

Mean pollutant concentrations (µg/m^3^) are predicted by FARM (Flexible Air quality Regional Model) for the entire study period at the municipality level (see legends for concentration values). The FARM model is a chemical–physical model of air quality that simulates the dispersion and chemical reactions of atmospheric pollutants. It integrates the data measured from the monitoring stations of the ARPA Lombardy air quality network, meteorological data, emissions, concentrations at the beginning of the simulation period, and trends in adjacent areas, covering the whole Lombard territory. The distribution of patients is reported as georeferenced to their residential address (dots).

### 3.2. Association between Air Pollutants and RA Disease Activity

#### 3.2.1. Fine Particulate Matter (PM_2.5_)

Regarding the association between PM_2.5_ and RA disease activity (DAS28 and SDAI) in the week preceding the visit, no statistically significant changes were observed in the multivariable linear regression models (Table 2).

A statistically significant association was observed for PhGA and the PM_2.5_ concentration the day of the evaluation (day-0), the day before (day-1), and 4 and 5 days (day-4 and -5) before the evaluation. Regarding TJC, a statistically significant change was observed only for PM_2.5_ concentration 5 days before the evaluation (day-5) (Appendix A, Table A5).

**Figure A1:**
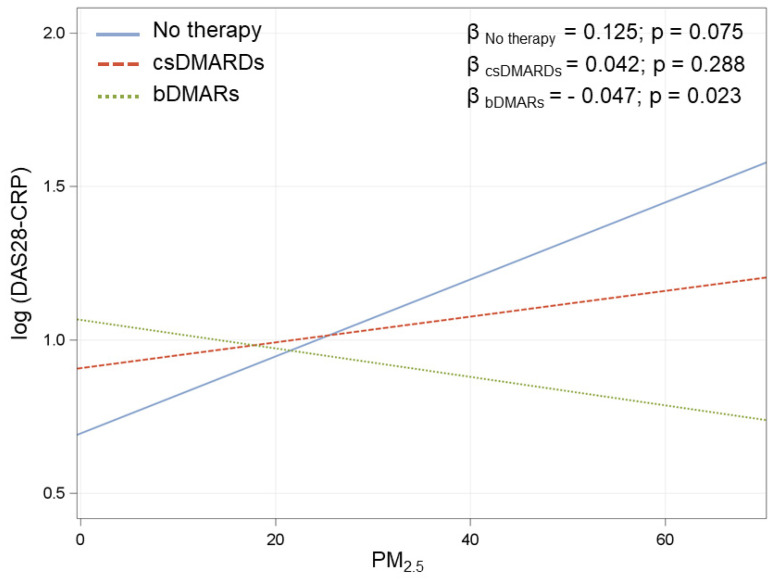
Association between PM_2.5_ at day 0 and DAS28 in the three treatment groups. β coefficients are reported for 10 µg/m^3^ increments in PM_2.5_. IC: Interval Confidence. Multivariate model adjusted for radiological damage, smoke, seropositivity, treatment (3 categories), corticosteroids, age at visit, disease duration, and interaction between PM_2.5_ and treatment.

When therapy interaction was added, a significant interaction was found for PM_2.5_ at the day of the visit (day-0), resulting in an inverse association between PM_2.5_ and DAS28 for patients with b/tsDMARD and no significant association for patients without ongoing therapy or those treated with csDMARDs (Table 2 and Appendix A, Figure A1). Some therapy interactions were also found for GH (day of evaluation), PaGA (day of evaluation), PhGA (7 days before the evaluation), and SJC (2 and 7 days before the evaluation) (Appendix A, Table A5).

#### 3.2.2. Particulate Matter (PM_10_)

Considering the association between PM_10_ and RA disease activity (DAS28 and SDAI), a statistically significant change was observed only for DAS28 and the PM_10_ concentration 5 days before the evaluation (day-5) (*p* = 0.034) (Table 3). PhGA and TJC showed scattered statistically significant associations with PM_10_. PhGA was found associated with PM_10_ 1, 4, 5 days before the evaluation (day-1, -4, and -5) and also for the whole week (week-1). Regarding TJC, a statistically significant change was observed only for PM_10_ concentration on the day of the evaluation (day-0) (Appendix A, Table A6).

The association between PM_10_ and RA disease activity (DAS28 and SDAI) was not modified by therapy (Table 3). Conversely, some therapy interactions were found for PhGA (6 and 7 days before the evaluation) and SJC (2 and 7 days before the evaluation) (Appendix A, Table A6).

#### 3.2.3. Nitrogen Dioxide (NO_2_)

Association between NO_2_ and RA disease activity (DAS28 and SDAI) was significant on the day of evaluation (day-0) and the day before (day-1) (Table 4). Only PhGA showed a statistically significant association with NO_2_ on the day before and 2 days before the evaluation (Appendix A, Table A7).

When therapy interaction was added, a significant interaction was found for PM_2.5_ on the day of the visit (day-0) and the day before the visit (day-1) for DAS28, but not for SDAI (Table 4). Regarding the other parameters (GH, PaGA, PhGA, SJC, TJC) no therapy interactions were observed.

#### 3.2.4. Ozone (O_3_)

O_3_ depicted an opposite trend. Association between O_3_ and RA disease activity (DAS28 and SDAI) was significant the day before the evaluation (day-0) for both indexes and 5 days before the evaluation (day-5) for DAS28 (Table 5).

GH, PhGA, and TJC showed a sparse statistically significant association with O_3_. A statistically significant association was observed for GH and the O_3_ concentration the day before the evaluation (day-1). Some associations (day-0, day-1, day-5, and week-1) were found between O_3_ concentration and PhGA. Regarding TJC, a statistically significant change was observed only for O_3_ concentration 5 days before the evaluation (day-5) (Appendix A, Table A8).

**Figure A2:**
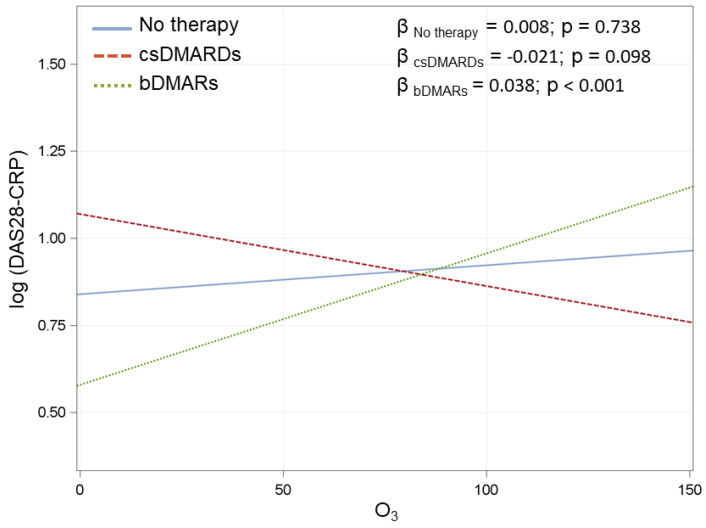
Association between O_3_ at day-1 and DAS28-CRP in the three treatment groups. β coefficients are reported for 10 µg/m^3^ increments in O_3_. IC–Interval Confidence. Multivariate model adjusted for radiological damage, smoke, seropositivity, treatment (3 categories), corticosteroids, age at visit, disease duration, and interaction between O_3_ and treatment.

An interaction of O_3_ with therapy was found for almost every day for DAS28 while only for the three days before the evaluation (day-1, -2, and -3) for SDAI. A significant therapy interaction was found for all the days for PhGA. Some therapy interactions were also found for GH (1 day before the evaluation) and TJC (1 and 7 days before the evaluation) (Table 5 and Appendix A, Table A8 and Figure A2).

## 4. Discussion

Disease activity in our Italian cohort of RA patients was not significantly affected by short-term air pollutants exposure in urban and peri-urban areas in the Lombardy region in the North of Italy. As can be observed in the maps (Figure 1), in these areas air pollution is particularly elevated due to human activities and geography. Notably, although scattered statistically significant associations were observed between short-term exposure to outdoor air pollutants (PM_10_, PM_2.5_, NO_2_, and O_3_) and RA activity, the changes did not reach the minimal clinically important difference [22].

A clear comparison with the other existing studies on this topic is hardly feasible because of the differences in methodology [16,17]. First, our included RA patients were in remission or had low disease activity (Table 1). By contrast, in the study of Wu, hospital re-admissions within one year were considered, thus suggesting more severe flares of disease [17]. The Kuwaiti study described an association between DAS28, clinical disease activity index (CDAI), NO_2,_ and SO_3_ using data from the national registry [16]. This latter study, as well as our results, provided very small variations in the outcome measures (i.e., disease activity), which, even if statistically significant, did not reach the minimal clinically important difference [22]. Finally, due to the differences in study design (cross-sectional vs. longitudinal case-crossover study) and in the period of air pollution exposure before the clinical assessment (seven days “short-term” vs. 30-day and 60-day medium-term), this study is not comparable with the other recent Italian study [18].

These findings are consistent with the previous studies on cigarette smoking exposure that is recognized as one of the most influencing environmental factors for RA susceptibility, but there is no evidence of its short-term influence on disease activity [23]. Moreover, our results are in line with those on SLE disease activity, which failed to prove the association between SLEDAI-2K and PM_2.5_ concentrations [24].

Notably, a strength of our study was the large number of subjects recruited, which allowed us to consider the possible role of the ongoing therapy as an effect modifier. Therapy seemed able to influence the relationship between short-term air pollution exposure and RA disease activity. It should be noted that this result is limited to PM_2.5_ levels and DAS28 at the day of the visit and O_3_ levels and disease activity scores (DAS28 and SDAI) for several days concerning the three groups of therapies.

Our study has some limitations: household and workplace air pollution were not considered, data on patients’ jobs, activities, urbanization, and socio-economic status were not collected. Moreover, this is a single-center study, and data are limited to our tertiary referral center: the study population was constituted of RA patients with long-standing disease and low disease activity. Lastly, air pollutants were considered as daily means while for some of them (e.g., O_3_), eight-hour averages would have been better than the daily mean.

As already mentioned, much of our current understanding of the impact of air pollution on RA pathobiological events has been derived from long-term retrospective studies using registries, administrative databases, or in vitro studies [4,5,13]. Although the environment seems to play a crucial role in inducing autoimmunity, it seems barely relevant for the short-term exposure to disease activity once the loss of tolerance is established in rheumatologic disorders (i.e., RA and SLE).

## 5. Conclusions

The conclusions of this study highlight the barely relevant contribution of short-term air pollutant exposure as triggering factors for RA flare. Previous findings favor a hypothesis that long-term exposure to environmental pollutants can induce autoimmunity, while the role of short- and medium-term exposure as potential contextual factors has not yet been clarified. Despite the strong in vitro evidence that particulate matter enhances the inflammatory pathways, the evidence of this effect in real-life RA patients is still a matter of debate. A clear comparison with the other existing studies on this topic is hardly feasible because of the differences in methodology for study designs (prospective versus retrospective), data source (registry, administrative database, or single-center cohort) and period of exposure (long-medium-term exposure versus short-term).

Future studies need to capture RA flares most accurately across different areas, taking into account both outdoor and indoor air pollution exposure, in addition to any additional health problems that may be induced by disease exacerbation and direct and indirect costs. Further multicenter research on different periods (long-, medium- and short-term) of exposure shall help to reveal determinants of RA flares to improve disease management. This will allow us to depict a more accurate picture of the burden of air pollution on RA.

## Figures and Tables

**Table 1:** Demographic and disease patient characteristics.

	Total	No DMARDs (n = 25)	csDMARDs (n = 108)	b/tsDMARDs (n = 289)
Age (years), mean (SD)	(n = 422)	61.1 (13.7)	56 (13.8)	58.8 (13)
Females, n (%)	58.2 (13.3)	20 (80)	86 (80)	238 (82)
BMI, mean (SD)	344 (81.5)	25.3 (3.6)	24.3 (4.6)	24 (4.5)
Current smokers, n (%)	24.1 (4.5)	5 (20)	14 (13)	89 (31)
Past smokers, n (%)	108 (27.3)	7 (28)	37 (34)	70 (24)
Disease duration, (yrs), mean (SD)	114 (28.9)	19.8 (19.5)	14.3 (11.8)	16.4 (10.3)
RF positivity, n (%)	16.1 (11.5)	12 (41.4)	52 (49.1)	187 (62.5)
ACPA positivity, n (%)	251 (59.5)	10 (34.5)	49 (46.2)	171 (59.6)
Corticosteroids, n (%)	230 (54.5)	8 (32)	59 (55)	137 (47)
DAS28-CRP, median (IQR)	204 (48.3)	2 (1.6–3.7)	2.3 (1.7–3.2)	2.3 (1.6–3.2)
SDAI, median (IQR)	2.3 (1.6–3.2)	6 (3.7–16.3)	5.8 (3.1–12.1)	6 (3–11.8)
GH (0–100), median (IQR)	6 (3–12)	60 (50–80)	70 (55–86)	70 (50–85)
PaGA (0–10), median (IQR)	70 (50–85)	4 (2–5)	3 (1.5–4.5)	3 (1.5–5)
PhGA (0–10) median (IQR)	3 (1.5–5)	2 (0–5)	1 (0–4)	1 (0–3)
ESR, (mm/h), median (IQR)	1 (0–4)	14 (8–27)	16 (8–30)	12 (5–28)
CRP, (mg/L), median (IQR)	14 (7–28)	2.1 (0.6–6.6)	2 (0.4–5)	2.8 (0.6–9)
SJC, median (IQR)	2.2 (0.5–7)	0 (0–0)	0 (0–1)	0 (0–1)
TJC, median (IQR)	0 (0–1)	0 (0–4)	1 (0–2)	0 (0–2)

ACPA: anti-citrullinated protein antibody; bDMARDs: biological-DMARDs; BMI: body mass index; csDMARDs: conventional synthetic Disease Modifying anti-Rheumatic Drugs; DAS28-CRP: Disease Activity Score in 28 joints using C-reactive protein; ESR: erythrocyte; GH: general health; IQR: interquartile range; PaGA: patient’s general assessment; PhGA: physician’s general assessment; RF: rheumatoid factor; SD: standard deviation; SDAI: Simplified Disease Activity Index; tsDMARDs: targeted synthetic-DMARDs; yrs: years.

**Table 2:** Associations between PM_2.5_ short-term exposure and RA disease activity.

PM_2.5_ Lag	DAS28-CRP		SDAI	
Day of Exposure	Estimate (SE)	DAS28-CRP	Estimate (SE)	*p*-Value
Day 0	−0.018 (0.018)	Estimate (SE)	−0.051 (0.050)	0.312
Day-1	−0.017 (0.018)	−0.018 (0.018)	−0.028 (0.051)	0.590
Day-2	−0.005 (0.019)	−0.017 (0.018)	−0.023 (0.054)	0.665
Day-3	−0.007 (0.015)	−0.005 (0.019)	−0.013 (0.043)	0.758
Day-4	−0.012 (0.015)	−0.007 (0.015)	0.001 (0.042)	0.983
Day-5	−0.031 (0.017)	−0.012 (0.015)	−0.050 (0.047)	0.288
Day-6	0.005 (0.017)	−0.031 (0.017)	0.044 (0.047)	0.348
Day-7	0.007 (0.016)	0.005 (0.017)	0.046 (0.047)	0.327
Week-1	−0.021 (0.022)	0.007 (0.016)	−0.029 (0.063)	0.646
		−0.021 (0.022)		

Multivariate model adjusted for radiographic damage, smoking status, seropositivity, therapy, steroid, age at visit, disease duration, and therapy. β coefficients are reported for 10 µg/m^3^ increments in PM_2.5_. Est.: estimate; SE–standard error; DAS28-CRP–Disease Activity Score in 28 joints using C-reactive protein; SDAI–Simplified disease activity index.

**Table 3:** Associations between PM_10_ short-term exposure and RA disease activity.

PM_10_ Lag	DAS28-CRP		SDAI	
Day of Exposure	Estimate (SE)	DAS28-CRP	Estimate (SE)	*p*-Value
Day 0	−0.018 (0.015)		−0.023 (0.042)	0.580
Day-1	−0.020 (0.015)	−0.018 (0.015)	−0.010 (0.043)	0.809
Day-2	−0.010 (0.015)	−0.020 (0.015)	−0.021 (0.043)	0.624
Day-3	−0.006 (0.012)	−0.010 (0.015)	−0.004 (0.035)	0.910
Day-4	−0.014 (0.012)	−0.006 (0.012)	−0.007 (0.034)	0.840
Day-5	−0.028 (0.013)	−0.014 (0.012)	−0.041 (0.038)	0.276
Day-6	−0.001 (0.014)	−0.028 (0.013)	0.026 (0.039)	0.503
Day-7	−0.005 (0.014)	−0.001 (0.014)	0.017 (0.039)	0.672
Week-1	−0.025 (0.018)	−0.005 (0.014)	−0.019 (0.052)	0.708

Multivariate model adjusted for radiographic damage, smoking status, seropositivity, therapy, steroid, age at visit, disease duration, and therapy. β coefficients are reported for 10 µg/m^3^ increments in PM_10_. Est.: estimate; SE–standard error; DAS28-CRP–Disease Activity Score in 28 joints using C-reactive protein; SDAI–Simplified disease activity index.

**Table 4:** Associations between NO_2_ short-term exposure and RA disease activity.

NO_2_ Lag	DAS28-CRP		SDAI	
Day of Exposure	Estimate (SE)	*p*-Value	Estimate (SE)	*p*-Value
Day 0	−0.027 (0.013)	0.038 *	−0.065 (0.037)	0.085
Day-1	−0.041 (0.014)	0.004 *	−0.069 (0.040)	0.088
Day-2	−0.019 (0.014)	0.170	−0.031 (0.040)	0.444
Day-3	−0.009 (0.013)	0.521	0.003 (0.038)	0.933
Day-4	−0.019 (0.013)	0.149	−0.014 (0.037)	0.704
Day-5	−0.014 (0.014)	0.295	−0.011 (0.038)	0.780
Day-6	−0.016 (0.014)	0.243	−0.019 (0.039)	0.631
Day-7	−0.015 (0.014)	0.273	−0.018 (0.038)	0.637
Week-1	−0.027 (0.016)	0.083	−0.038 (0.045)	0.398

Multivariate model adjusted for radiographic damage, smoking status, seropositivity, therapy, steroid, age at visit, disease duration, and therapy. β coefficients are reported for 10 µg/m^3^ increments in NO_2_. Est.: estimate; SE–standard error; DAS28-CRP–Disease Activity Score in 28 joints using C-reactive protein; SDAI–Simplified disease activity index. * Statistically significant influence of therapy on the interaction between NO_2_ and disease activity.

**Table 5:** Associations between O_3_ short-term exposure and RA disease activity.

O_3_ Lag	DAS28-CRP		SDAI	
Day of Exposure	Estimate (SE)	*p*-Value	Estimate (SE)	*p*-Value
Day 0	0.110 (0.070)	0.124 *	0.320 (0.200)	0.108
Day-1	0.018 (0.007)	0.011 *	0.043 (0.020)	0.031 *
Day-2	0.010 (0.007)	0.132 *	0.026 (0.019)	0.166 *
Day-3	0.010 (0.006)	0.123 *	0.019 (0.018)	0.284 *
Day-4	0.009 (0.007)	0.169	0.012 (0.019)	0.508
Day-5	0.016 (0.007)	0.014 *	0.031 (0.019)	0.099
Day-6	0.009 (0.007)	0.194 *	0.017 (0.020)	0.410
Day-7	0.009 (0.007)	0.247 *	0.008 (0.021)	0.708
Week-1	0.015 (0.007)	0.051 *	0.033 (0.021)	0.120

Multivariate model adjusted for radiographic damage, smoking status, seropositivity, therapy, steroid, age at visit, disease duration, and therapy. β coefficients are reported for 10 µg/m^3^ increments in O_3_. Est.: estimate; SE–standard error; DAS28-CRP–Disease Activity Score in 28 joints using C-reactive protein; SDAI–Simplified disease activity index. * Statistically significant influence of therapy on the interaction between O_3_ and disease activity.

## Data Availability

The data underlying this article will be shared at reasonable request to the corresponding author. Data of the Regional Environmental Protection Agency are available at www.arpalombardia.it/Pages/Aria/Modellistica/I-sistemi-modellistici-in-ARPA.aspx (accessed 10 August 2021).

## Appendix A

**Table A1:** PM_2.5_ concentrations in the 1 week before the evaluation.

PM_2.5_ Lag	Mean	SD	Median	Lower Quartile	Upper Quartile	Min	Max
Day 0	28.5	12.5	25.4	19.3	36.5	4.5	68.4
Day-1	27	12	23.9	18	35.6	4.4	66.4
Day-2	27	11.6	25.8	18.6	35.1	4.8	71.8
Day-3	28.9	14.5	26.4	17.7	38.1	5.3	83
Day-4	29.8	14.8	26.8	18.9	37.9	4.2	74.8
Day-5	28.4	13.2	26.3	18.8	36.5	3.2	71.3
Day-6	28.2	13.2	25.7	18.4	35.6	5.6	74.3
Day-7	27.7	13.4	24.3	18	35.6	6.2	67.5
Week-1	28.3	9.9	28	21	35	6	62

SD–standard deviations; IQR—interquartile range.

**Table A2:** PM_10_ concentrations in the 1 week before the evaluation.

PM_10_ Lag	Mean	SD	Median	Lower Quartile	Upper Quartile	Min	Max
Day 0	35.7	14.9	33	24.9	46.2	6.6	88.1
Day-1	33.1	14.4	31.1	23.3	43.2	5.1	76.2
Day-2	33	14.5	31.2	22.7	42.2	5.1	91.6
Day-3	35	17.7	32.5	21.9	46.8	3.7	95.7
Day-4	36.1	18.4	32.1	22.8	46.9	4.3	96
Day-5	35	16.6	31.8	22.5	45.1	3.7	95.6
Day-6	35.1	16	32	23.7	43.5	6	90.4
Day-7	34.1	15.7	30.6	23.1	42.7	6.6	86.2
Week-1	34.8	12	34	26	42	7	68

SD–standard deviations; IQR—interquartile range.

**Table A3:** NO_2_ concentrations in the 1 week before the evaluation.

NO_2_ Lag	Mean	SD	Median	Lower Quartile	Upper Quartile	Min	Max
Day 0	44.3	16.7	46.3	31	56.6	4.4	84.3
Day-1	40.8	15.5	41.9	30.1	51.9	3.6	76.9
Day-2	40.7	15.8	41.5	29.4	52.4	2.9	95.4
Day-3	42.3	16.4	42.6	30.4	53.9	3.1	91.7
Day-4	43.8	16.8	45.6	32.7	55.1	2.6	95.4
Day-5	44.4	16.4	47.6	33.5	55.8	4.5	84.1
Day-6	45.1	16.5	47.9	34.3	57.3	5.9	95.4
Day-7	43.1	16.5	44.8	31	55.1	4.1	85.8
Week-1	43.1	14.1	45	34	54	5	83

SD–standard deviations; IQR–interquartile range.

**Table A4:** O_3_ concentrations in the 1 week before the evaluation.

O_3_ Lag	Mean	SD	Median	Lower Quartile	Upper Quartile	Min	Max
Day 0	59.2	32	51.3	37.5	75.2	9.8	192
Day-1	61	32.2	55.1	38.6	76.3	9.4	170.5
Day-2	61	34	54.1	35.7	78.2	9.8	169.7
Day-3	60.3	34.3	53	33.4	80.5	9.4	167.4
Day-4	57.8	33.3	49.1	32.2	76.1	8.2	163.2
Day-5	58.9	32.6	54.2	35.7	74.6	12.9	175
Day-6	57.3	30.9	52.5	34	75.3	14.7	172.5
Day-7	57.1	30	53	36.8	72.4	8.2	184.1
Week-1	59.2	29.6	50	37	76	22	168

SD–standard deviations; IQR–interquartile range.

**Table A5:** Associations between PM_2.5_ short-term exposure and RA disease activity.

PM_2.5_ Lag	GH		PaGA		PhGA		SJC		TJC	
Day of Exposure.	Estimate (SE)	*p*-Value	Estimate (SE)	*p*-Value	Estimate (SE)	*p*-Value	Estimate (SE)	*p*-Value	Estimate (SE)	*p*-Value
Day 0	−0.006 (0.017)	0.717 *	0.005 (0.03)	0.878 *	−0.102 (0.036)	0.005	−0.058 (0.062)	0.349	−0.089 (0.049)	0.075
Day-1	0.016 (0.017)	0.359	−0.006 (0.03)	0.850	−0.112 (0.035)	0.002	0.061 (0.064)	0.336	−0.066 (0.051)	0.197
Day-2	−0.003 (0.018)	0.859	0.022 (0.032)	0.493	−0.046 (0.039)	0.234	0.057 (0.066)	0.391 *	−0.024 (0.054)	0.654
Day-3	−0.01 (0.015)	0.506	0.024 (0.025)	0.341	−0.03 (0.031)	0.341	0.048 (0.051)	0.345	−0.018 (0.041)	0.667
Day-4	−0.021 (0.014)	0.142	0.027 (0.025)	0.279	−0.064 (0.029)	0.030	0.012 (0.051)	0.817	−0.051 (0.041)	0.216
Day-5	0.002 (0.016)	0.901	−0.023 (0.028)	0.417	−0.115 (0.032)	0.001	−0.028 (0.06)	0.637	−0.100 (0.049)	0.041
Day-6	−0.026 (0.016)	0.103	0.010 (0.028)	0.727	−0.054 (0.033)	0.106	0.03 (0.059)	0.616	−0.037 (0.047)	0.429
Day-7	−0.019 (0.016)	0.238	−0.019 (0.027)	0.489	−0.021 (0.033)	0.530 *	−0.024 (0.053)	0.652 *	−0.019 (0.045)	0.678
Week-1	−0.014 (0.022)	0.508	0.018 (0.038)	0.624	−0.135 (0.045)	0.003	0.035 (0.080)	0.667	−0.096 (0.064)	0.134

Multivariate model adjusted for radiographic damage, smoking status, seropositivity, therapy, steroid, age at visit, disease duration, and therapy. β coefficients are reported for 10 µg/m^3^ increments in PM_2.5_. LCL–lower confidence level; UCL—upper confidence level; SE–standard error; DAS28-CRP–Disease Activity Score in 28 joints using C-reactive protein; GH–general health; PaGA—patient’s general assessment; PhGA—physician’s general assessment; SJC—swollen joint count; TJC—tender joint count. *—Statistically significant interaction between PM_2.5_ and therapy on disease activity.

**Table A6:** Associations between PM_10_ short-term exposure and RA disease activity.

PM_10_ Lag	GH		PaGA		PhGA		SJC		TJC	
Day of Exposure.	Estimate (SE)	*p*-Value	Estimate (SE)	*p*-Value	Estimate (SE)	*p*-Value	Estimate (SE)	*p*-Value	Estimate (SE)	*p*-Value
Day 0	−0.010 (0.014)	0.475	0.005 (0.025)	0.842	−0.073 (0.03)	0.017	−0.054 (0.053)	0.313	−0.085 (0.042)	0.044
Day-1	0.006 (0.015)	0.674	0.006 (0.025)	0.828	−0.091 (0.03)	0.003	0.021 (0.054)	0.696	−0.058 (0.043)	0.178
Day-2	−0.005 (0.015)	0.744	0.014 (0.025)	0.572	−0.041 (0.03)	0.177	0.027 (0.05)	0.589 *	−0.021 (0.042)	0.622
Day-3	−0.012 (0.012)	0.299	0.023 (0.021)	0.273	−0.015 (0.025)	0.553	0.032 (0.041)	0.442	−0.017 (0.034)	0.614
Day-4	−0.015 (0.011)	0.192	0.011 (0.02)	0.573	−0.052 (0.024)	0.028	0.011 (0.042)	0.789	−0.033 (0.034)	0.335
Day-5	0.003 (0.013)	0.833	−0.031 (0.022)	0.169	−0.088 (0.026)	0.001	−0.043 (0.048)	0.380	−0.063 (0.04)	0.120
Day-6	−0.019 (0.013)	0.152	0.003 (0.023)	0.896	−0.052 (0.027)	0.058 *	0.03 (0.049)	0.538	−0.008 (0.039)	0.836
Day-7	−0.007 (0.013)	0.616	−0.038 (0.023)	0.105	−0.035 (0.028)	0.224 *	−0.031 (0.047)	0.503 *	−0.011 (0.039)	0.777
Week-1	−0.015 (0.018)	0.401	0.009 (0.031)	0.770	−0.102 (0.036)	0.006	0.015 (0.065)	0.816	−0.069 (0.053)	0.196

Multivariate model adjusted for radiographic damage, smoking status, seropositivity, therapy, steroid, age at visit, disease duration, and therapy. β coefficients are reported for 10 µg/m^3^ increments in PM_10_. LCL—lower confidence level; UCL—upper confidence level; SE—standard error; DAS28-CRP—Disease Activity Score in 28 joints using C-reactive protein; GH—general health; PaGA—patient’s general assessment; PhGA–physician’s general assessment; SJC—swollen joint count; TJC—tender joint count. *—Statistically significant interaction between PM_10_ and therapy on disease activity.

**Table A7:** Associations between NO_2_ short-term exposure and RA disease activity.

NO_2_ lag	GH		PaGA		PhGA		SJC		TJC	
Day of Exposure.	Estimate (SE)	*p*-Value	Estimate (SE)	*p*-Value	Estimate (SE)	*p*-Value	Estimate (SE)	*p*-Value	Estimate (SE)	*p*-Value
Day 0	0.013 (0.013)	0.303	−0.02 (0.022)	0.356	−0.051 (0.027)	0.055	−0.045 (0.042)	0.287	−0.033 (0.037)	0.372
Day-1	0.014 (0.014)	0.305	−0.02 (0.024)	0.396	−0.080 (0.028)	0.005	−0.042 (0.047)	0.367	−0.058 (0.041)	0.161
Day-2	0.012 (0.014)	0.359	−0.005 (0.024)	0.817	−0.058 (0.028)	0.038	−0.040 (0.043)	0.348	−0.036 (0.037)	0.335
Day-3	0.001 (0.013)	0.981	0.018 (0.022)	0.430	−0.030 (0.025)	0.231	−0.025 (0.041)	0.542	−0.029 (0.035)	0.407
Day-4	0.006 (0.013)	0.617	−0.015 (0.022)	0.508	−0.042 (0.026)	0.107	−0.060 (0.041)	0.148	−0.063 (0.037)	0.096
Day-5	0.006 (0.013)	0.651	−0.023 (0.023)	0.301	−0.032 (0.026)	0.215	−0.053 (0.041)	0.192	−0.046 (0.038)	0.222
Day-6	0.005 (0.013)	0.686	−0.018 (0.023)	0.430	−0.023 (0.027)	0.398	−0.023 (0.045)	0.610	−0.011 (0.039)	0.773
Day-7	0.004 (0.013)	0.758	−0.021 (0.022)	0.347	−0.029 (0.027)	0.287	−0.038 (0.043)	0.377	−0.012 (0.038)	0.761
Week-1	0.011 (0.015)	0.477	−0.017 (0.026)	0.524	−0.058 (0.030)	0.057	−0.052 (0.048)	0.282	−0.052 (0.044)	0.234

Multivariate model adjusted for radiographic damage, smoking status, seropositivity, therapy, steroid, age at visit, disease duration, and therapy. β coefficients are reported for 10 µg/m^3^ increments in NO_2_. LCL—lower confidence level; UCL—upper confidence level; SE—standard error; DAS28-CRP—Disease Activity Score in 28 joints using C-reactive protein; GH—general health; PaGA—patient’s general assessment; PhGA—physician’s general assessment; SJC—swollen joint count; TJC—tender joint count. Statistically significant interaction between NO_2_ and therapy on disease activity.

**Table A8:** Associations between O_3_ short-term exposure and RA disease activity.

Max O3 Lag	GH		PaGA		PhGA		SJC		TJC	
Day of Exposure.	Estimate (SE)	*p*-Value	Estimate (SE)	*p*-Value	Estimate (SE)	*p*-Value	Estimate (SE)	*p*-Value	Estimate (SE)	*p*-Value
Day 0	−0.012 (0.007)	0.085	0.010 (0.012)	0.415	0.033 (0.014)	0.017 *	0.027 (0.021)	0.201	0.03 (0.019)	0.109
Day-1	−0.016 (0.007)	0.021 *	0.019 (0.012)	0.106 *	0.030 (0.014)	0.035 *	0.028 (0.021)	0.191	0.029 (0.019)	0.133 *
Day-2	−0.008 (0.006)	0.191	0.012 (0.011)	0.274	0.017 (0.013)	0.188 *	0.032 (0.02)	0.118	0.020 (0.018)	0.268
Day-3	−0.007 (0.006)	0.276	0.002 (0.011)	0.828	0.023 (0.013)	0.073 *	0.021 (0.02)	0.298	0.018 (0.018)	0.325
Day-4	−0.001 (0.006)	0.916	−0.003 (0.011)	0.776	0.024 (0.013)	0.070 *	0.030 (0.021)	0.162	0.036 (0.018)	0.056
Day-5	−0.007 (0.007)	0.311	0.014 (0.011)	0.212	0.031 (0.013)	0.021 *	0.033 (0.021)	0.116	0.037 (0.018)	0.045
Day-6	−0.003 (0.007)	0.664	0.012 (0.012)	0.323	0.023 (0.014)	0.106 *	0.038 (0.021)	0.078	0.035 (0.019)	0.066
Day-7	−0.006 (0.007)	0.376	0.013 (0.012)	0.295	0.016 (0.014)	0.264 *	0.035 (0.022)	0.109	0.025 (0.020)	0.193 *
Week-1	−0.010 (0.007)	0.191	0.011 (0.012)	0.380	0.031 (0.015)	0.035 *	0.037 (0.022)	0.106	0.034 (0.020)	0.091

Multivariate model adjusted for radiographic damage, smoking status, seropositivity, therapy, steroid, age at visit, disease duration, and therapy. β coefficients are reported for 10 µg/m^3^ increments in O_3_. LCL—lower confidence level; UCL—upper confidence level; SE—standard error; DAS28-CRP—Disease Activity Score in 28 joints using C-reactive protein; GH–general health; PaGA—patient’s general assessment; PhGA—physician’s general assessment; SJC—swollen joint count; TJC—tender joint count. *—Statistically significant interaction between O_3_ and therapy on disease activity.
